# Hsa_circ_0003732 promotes osteosarcoma cells proliferation via miR-545/CCNA2 axis

**DOI:** 10.1042/BSR20191552

**Published:** 2020-06-23

**Authors:** Li Li, Xiang-an Kong, Mousheng Zang, Jisheng Dong, Yingqi Feng, Binjie Gui, Yong Hu

**Affiliations:** 1 Department of orthopedics, The First Affiliated Hospital of Anhui Medical University, Hefei, Anhui, China; 2 Department of orthopedics, The Second People's Hospital of Hefei, Hefei, Anhui, China

**Keywords:** CCNA2, Circular RNA, miR-545, Osteosarcoma

## Abstract

Osteosarcoma (OS) is a primary malignant bone tumor with a high fatality rate. Circular RNAs (circRNAs) are a type of endogenous noncoding RNA that have been verified to participate in cancer pathophysiological processes. We aim to investigate the roles of circRNAs in osteosarcoma tumorigenesis. In the present study, we showed that hsa_circ_0003732 was up-regulated in OS tissues and elevated level of hsa_circ_0003732 was linked to poor prognosis of OS patients. Functional investigation indicated that hsa_circ_0003732 promoted proliferation of OS cells. Furthermore, we identified miR-545 as the hsa_circ_0003732-associated microRNA and CCNA2 was a direct target of miR-545. In addition, hsa_circ_0003732 could elevate CCNA2 expression via miR-545, resulting in the promotion of OS cells proliferation. Altogether, our findings demonstrate that hsa_circ_0003732 promotes OS cells proliferation via miR-545/CCNA2 axis and imply hsa_circ_0003732 may be a potential prognosis biomarker and therapeutic target for OS.

## Introduction

Osteosarcoma (OS) is the most common bone malignancy that usually occurs in young people [1]. Despite the combination of advanced treatments such as surgery, chemotherapy and radiotherapy, the prognosis of OS patients is still poor due to the rapid progression [2,3]. The poor prognosis of osteosarcoma is partially due to the lack of good molecular biomarkers at diagnosis and effective therapeutic targets in the treatment [4]. So, there is an urgent requirement to investigate new molecules that were involved in osteosarcoma tumorigenesis and to discover novel diagnosis biomarkers and therapeutic targets for osteosarcoma.

Circular RNAs (circRNAs) are a class of noncoding RNA that is characterized with a covalently closed loop structure [5]. CircRNAs have been reported to participate in cancer progression and act as biomarkers for the diagnosis and prognosis of many cancers [6–9]. What’s more, some studies showed that circRNAs were aberrantly express in OS and involved in osteosarcoma tumorigenesis [10–13]. Thus, circRNAs have potential as candidates for diagnostic biomarkers and therapeutic targets in patients with OS and studying circRNAs in osteosarcoma has important clinical significance.

In this study, we found the expression of hsa_circ_0003732 that derived from back-splicing of HUWE1 transcript was increased in OS tissues using microarray analysis. Hsa_circ_0003732 rank the first among the upregulated circRNAs and its roles in osteosarcoma remained unclear, so it was chosen for further validation. Then, we verified the expression of hsa_circ_0003732 in OS tissues and cell lines by quantitative real-time PCR (qRT-PCR) as well as explored its value in acting as a clinical biomarker. We further investigated the functions and mechanisms of hsa_circ_0003732 in OS *in vitro* and discussed the probability to be a therapeutic target for osteosarcoma.

## Materials and methods

### Patients and specimens

This study was approved by the Ethics Committee of the First Affiliated Hospital of Anhui Medical University and conformed to the Ethical Guidelines of the Declaration of Helsinki. Forty-six pairs of OS tissues and adjacent noncancerous tissues (ANTs) from primary osteosarcoma patients were collected from patients who underwent complete resection surgery at the Department of Orthopedics of the First Affiliated Hospital of Anhui Medical University. Written informed consent was obtained from all the participants.

### circRNA microarray

Total RNA was extracted from three pairs of OS tissues and ANTs and digested with Rnase R (Epicentre, Inc.) to increase the enrichment of circRNA. circRNA microArray was performed using circRNA chip provided by Arraystar containing the specific probes for human circRNA.

### Quantitative real-time PCR (qRT-PCR)

Total RNA was isolated using TRIzol reagent (Invitrogen, Carlsbad, CA, U.S.A.) and reverse-transcribed by PrimeScript RT reagent Kit (Takara). qRT-PCR was performed using SYBR Green qPCR Master Mix (Thermo Fisher Scientific). The expressions of hsa_circ_0003732, c-Myc and CCND1 were normalized to GAPDH expression. miR-545 expression was assessed using Hairpin-it™ miRNAs qPCR Quantitation Kit (GeneParma, Shanghai, China) and normalized to U6 expression. The primers were: hsa_circ_0003732, F: 3′- CAGCAATGGCTGCCAGAATTA-5′, R: 3′- TTCAATGGGGCGGTGTAAGG-5′; GAPDH, F: 5′-AATGGGCAGCCGTTAGGAAA-3′, R: 5′- TGAAGGGGTCATTGATGGCA-3′.

### Cell culture and transfection

The human osteosarcoma cell lines (MG-63, HOS, U2OS and Saos-2) and human osteoblastic cell line (hFOB1.19) were cultured in DMEM Medium (Gibco, CA, U.S.A.) containing 10% fetal bovine serum. Oligoribonucleotides were transfected into cells using Lipofectamine 2000 (Invitrogen). The siRNA sequences for hsa_circ_0003732 siRNA were, sense: 5′- AGUGUCUGAUAACAAAGGCUUTT-3′, antisense: 5′- AAGCCUUUGUUAUCAGACACUTT-3′.

### Cell Counting Kit-8 (CCK-8) assay

Cell Counting Kit-8 (CCK-8) assay was used to study cell proliferation. Cells were seeded in 96-well plates (2000 cells/well) and transfected with oligoribonucleotides. CCK-8 kit (Dojindo, Japan) was used at 24, 48 and 72 h after transfection. The optical density was measured by a microplate reader at 450 nm.

### Cell cycle assay

Cells were seeded in six-well plates and transfected with oligoribonucleotides. After 48 h, cells were harvested and cell cycle was analyzed by DNA Content Quantitation Assay (Cell Cycle) (Solarbio life sciences, Beijing, China) and Flow cytometry.

### 5-Ethynyl-2-deoxyuridine (EdU) assay

EdU assay was applied for detection of DNA replication activity. Cells were seeded in six-well plates and transfected with oligoribonucleotides for 48 h. A Cell-Light EdU Apollo567 In Vitro Kit (Ribobio, China) was employed to conduct the EdU assays. Hoechst staining was used for labeling living cells. Images were acquired with a fluorescence microscope (Olympus, Japan).

### Western blot Assays

Cells were lysed with RIPA (Beyotime, China) supplemented with protease inhibitor cocktail to extract proteins. Protein concentrations were measured with BCA protein assay Kit (BioRad, Hercules, CA, U.S.A.). Equal amounts of proteins were resolved on SDS-denaturing polyacrylamide gels, and transferred onto PVDF membranes (Millipore, Bedford, MA, U.S.A.). The primary antibodies used for Western blot were: CCNA2 antibody (#4656, Cell Signaling Technology). Protein levels were normalized to β-actin (AF5003, Beyotime, Shanghai, China).

### Dual luciferase reporter assay

The reporter plasmids were obtained by inserting wild-type or mutant hsa_circ_0003732 or CCNA2 3′UTR sequences that contained predicted miR-545 binding sites into the pmirGLO vector (Promega, Madison, U.S.A.). miR-545 mimics and reporter plasmids were co-transfected into cells and cultured for 48 h. Luciferase activity was detected using Dual-luciferase reporter assay system (Promega) and normalized to the Renilla luciferase internal control.

### Statistical analysis

Statistical analyses were performed with the SPSS 19.0 software (SPSS, IL, U.S.A.). Data are presented as the mean ± SD. Student’s *t*-test and one-way analysis of variance were applied to evaluate differences among groups. Chi-squared test was used to analyze the correlation of hsa_circ_0003732 expression with clinicopathological factors. Kaplan–Meier method and log-rank test was used to conduct survival analysis. *P*<0.05 was considered statistically significant. The independent experiments were performed for at least three replicates.

## Results

### Hsa_circ_0003732 expression was increased in OS tissues and correlated with poor prognosis of OS patients

**Figure 1:**
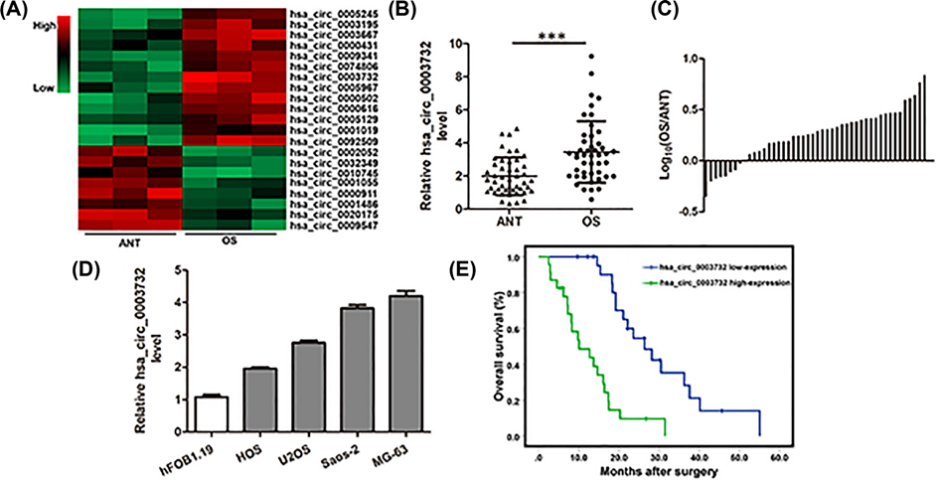
Hsa_circ_0003732 expression was increased in OS tissues and correlated with poor prognosis of OS patients (**A**) Heatmap showing the circRNA expression profiles of OS tissues and adjacent non-tumor tissues. (**B**) Relative expression of hsa_circ_0003732 in 46 pairs of OS tissues and ANTs were analyzed by qRT-PCR and normalized to GAPDH expression. Data were analyzed by the delta Ct method and compared with paired Student’s *t*-tests. (**C**) Relative hsa_circ_0003732 expression with the ratio of its level in OS tissues versus ANTs shown on the logarithmic scale. (**D**) Relative hsa_circ_0003732 expression detected by qRT-PCR in OS cell lines (HOS, U2OS, Saos-2 and MG-63) and human osteoblastic cell line (hFOB1.19). (**E**) Overall survival rate of OS patients with hsa_circ_0003732 high-expression and hsa_circ_0003732 low-expression; *P*<0.001. Data are presented as means ± S.D., ***P<0.001.

Through microarray analysis, we found that hsa_circ_0003732 expression was increased in OS tissues compared with ANTs (Figure 1A). In further experiments, we validated the up-regulation of hsa_circ_0003732 expression in 46 pairs of OS tissues and ANTs (Figure 1B,C). We also found that circ_0003732 expression was higher in OS cells compared with hFOB1.19 cells (Figure 1D). Subsequently, we classified the forty-six osteosarcoma patients into hsa_circ_0003732 high-expression and low-expression groups using the median hsa_circ_0003732 level as the cut-off. As shown in Table 1, compared with the hsa_circ_0003732 low-expression group, larger tumor size and advanced Enneking stage were observed in the hsa_circ_0003732 high-expression group. In addition, Kaplan–Meier analysis showed that patients of hsa_circ_0003732 high-expression group had lower overall survival rate compared with the hsa_circ_0003732 low-expression group (Figure 1E). These findings demonstrated hsa_circ_0003732 expression was increased in OS tissues and correlated with poor prognosis of OS patients.

**Table 1:** Relationship between clinical variables of OS patients and hsa_circ_0003732 level in OS tissues

Variables	*N*	hsa_circ_0003732 low-expression	hsa_circ_0003732 high-expression	*P* value
Gender				
Male	25	11	14	
Female	21	12	19	0.277
Age (years)				
<25	29	12	17	
≥25	17	11	6	0.111
Tumor size (cm)				
<6	32	20	12	
≥6	14	3	11	**0.012**
Enneking stage				
I+IIA	34	20	14	
IB+III	12	3	8	**0.045**
Lung metastasis				
No	35	20	15	
Yes	11	3	8	0.083
Location				
Femur/tibia	32	16	16	
Elsewhere	14	7	7	0.625
Differentiation grade				
Well/moderately	27	15	12	
Poorly/undifferentiated	19	8	11	0.275

Abbreviations: OS, osteosarcoma; hsa_circ_0003732 low/high-expression: the expression of hsa_circ_0003732 in OS tissues was lower/higher than the median.

### Knockdown of hsa_circ_0003732 inhibited proliferation of OS cells

**Figure 2:**
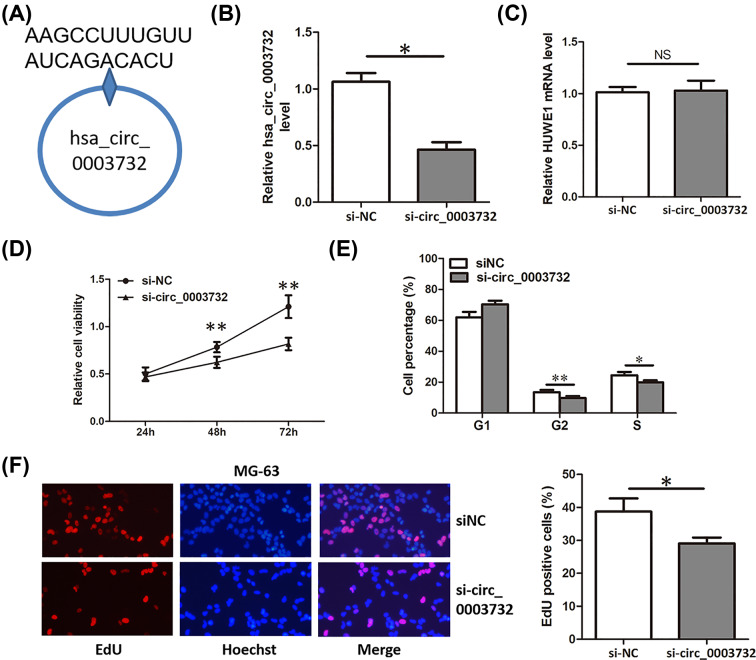
Knockdown of hsa_circ_0003732 inhibited proliferation of OS cells (**A**) Target site of hsa_circ_0007255 siRNAs in the back-splice junction. (**B** and **C**) Relative hsa_circ_0003732 and HUWE1 mRNA level in MG-63 cells transfected with hsa_circ_0003732 siRNA. (**D**) CCK8 assays showing proliferation of MG-63 cells transfected with hsa_circ_0003732 siRNA. (**E**) Cell cycle assays showing cell proportion of G2 and S phase in MG-63 cells transfected with hsa_circ_0003732 siRNA. (**F**) EdU assays assessing DNA replication activity of MG-63 cells transfected with hsa_circ_0003732 siRNA. Data are presented as means ± S.D., NS, no significance, *P<0.05, **P<0.01.

Because the expression of hsa_circ_0003732 was significantly higher in OS cells than that of hFOB1.19 cells (Figure 1C). Specific siRNA targeting hsa_circ_0003732 (Figure 2A) were transfected into MG-63 cells, leading to a significant down-regulation of hsa_circ_0003732 but not the HUWE1 mRNA (Figure 2B,C). We detected the proliferation of MG-63 cells transfected with hsa_circ_0003732 siRNA using CCK-8 assay, cell cycle assay and EdU assay. Compared with negative control, knockdown of hsa_circ_0003732 significantly decreased cell viability (Figure 2D), cell proportion of G2 and S phase (Figure 2E) and DNA replication activity (Figure 2F) in MG-63 cells. These results suggested that knockdown of hsa_circ_0003732 inhibited proliferation of OS cells.

### Hsa_circ_0003732 acted as a sponge for miR-545

**Figure 3:**
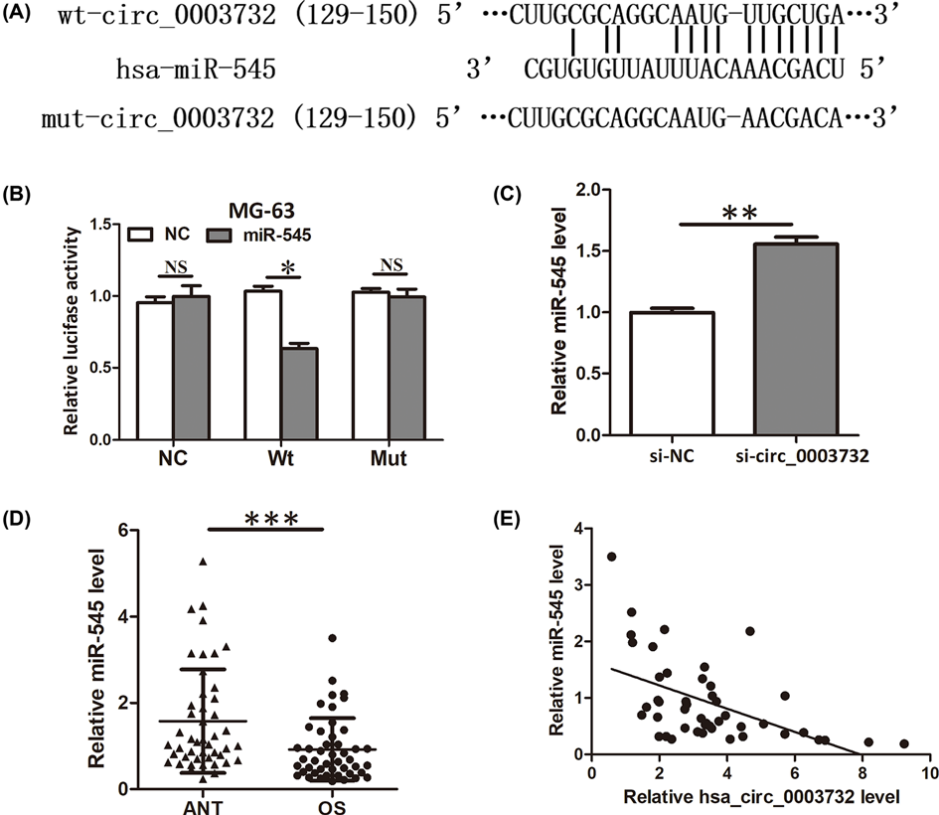
Hsa_circ_0003732 acted as a sponge for miR-545 (**A**) Diagram showing the binding of miR-545 and hsa_circ_0003732 wild-type (Wt) or mutant (Mut). (**B**) Luciferase reporter assay showed miR-545 overexpression reduced the luciferase activity of the hsa_circ_0003732 Wt reporter but not the hsa_circ_0003732 Mut reporter. (**C**) qRT-PCR showing miR-545 level when MG-63 cells were transfected with hsa_circ_0003732 siRNA. (**D**) Relative expression of miR-545 in 46 pairs of OS tissues and ANTs were analyzed by qRT-PCR and normalized to U6 expression. (**E**) Pearson’s correlation analysis indicated an inverse correlation between miR-545 level and hsa_circ_0003732 level in OS tissues (*r* = −0.527, *P*<0.001). Data are presented as means ± S.D., NS, no significance, *P<0.05, **P<0.01, ***P<0.001.

Then, we sought to explore the molecular mechanism of hsa_circ_0003732 in promoting proliferation of OS cells. Because circRNAs could act as a sponge to repress miRNA availability, so, we performed bioinformatics analysis to uncover the potential miRNAs that might binding to hsa_circ_0003732. We found hsa_circ_0003732 possessed a complementary sequence to miR-545 seed region (Figure 3A). Subsequently, luciferase reporter assay was performed to verify the interaction between hsa_circ_0003732 and miR-545. The results showed that miR-545 significantly inhibited the luciferase activity of MG-63 cells transfected with the reporter containing wild-type hsa_circ_0003732 sequence but not the reporter vector containing the mutant binding sites of hsa_circ_0003732 sequence (Figure 3B). Moreover, increased expression of miR-545 was observed when MG-63 cells were transfected with hsa_circ_0003732 siRNA (Figure 3C). Additionally, miR-545 expression was found decreased in OS tissues (Figure 3D) and negatively correlated with hsa_circ_0003732 expression (Figure 3E). These findings verified that hsa_circ_0003732 acted as a sponge for miR-545.

### MiR-545 inhibited proliferation of OS cells

**Figure 4:**
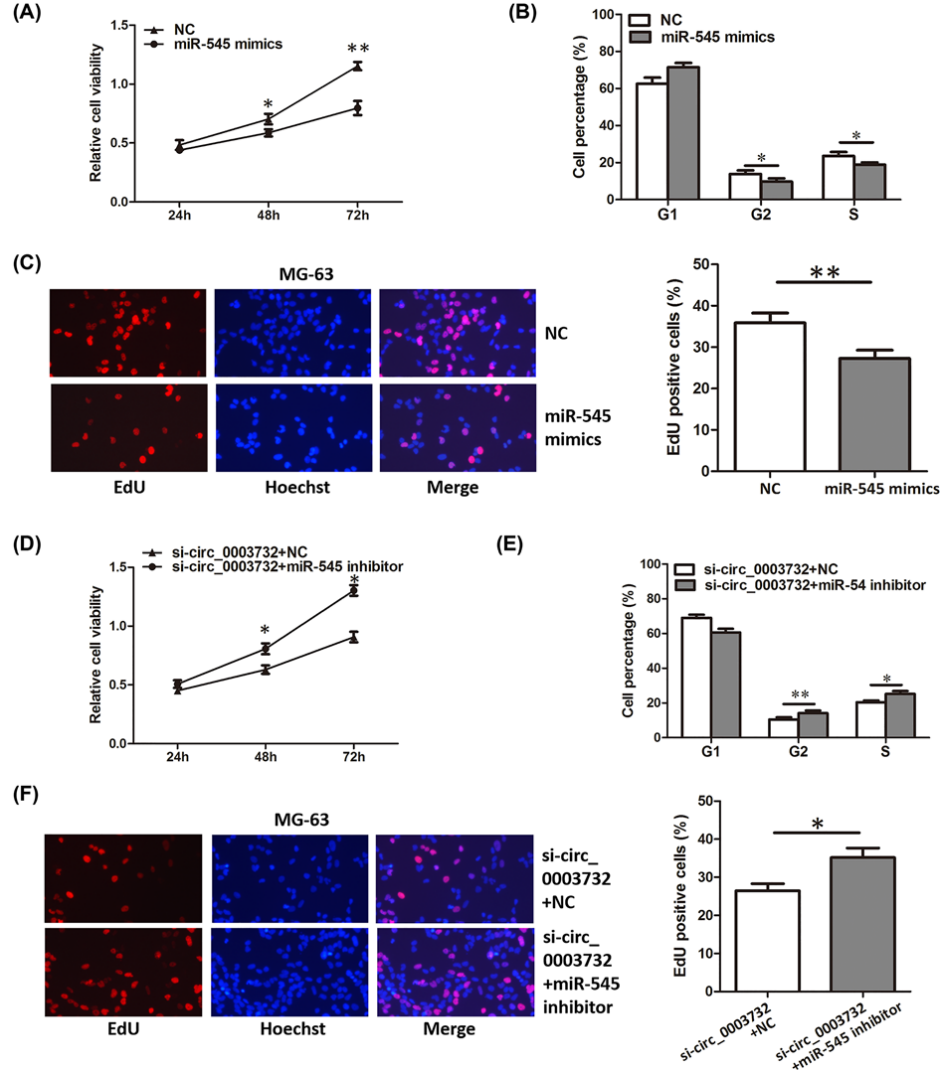
MiR-545 inhibited proliferation of OS cells (**A–C**) CCK8 assay, cell cycle assay and EdU assay showing proliferation of MG-63 cells transfected with miR-545 mimics. (**D–F**) CCK8 assay, cell cycle assay and EdU assay showing proliferation of MG-63 cells co-transfected with hsa_circ_0003732 siRNA and miR-545 inhibitor. Data are presented as means ± S.D., *P<0.05, **P<0.01.

Next, the role of miR-545 in OS was explored. Functional experiments showed that overexpression of miR-545 significantly inhibited the proliferation of OS cells (Figure 4A–C). In addition, miR-545 inhibitor could abrogate the proliferation inhibition induced by hsa_circ_0003732 knockdown (Figure 4D–F). These results demonstrated that miR-545 could inhibit proliferation and hsa_circ_0003732 regulated proliferation by sponging miR-545 in OS cells.

### CCNA2 was a direct target of miR-545 in OS cells

**Figure 5:**
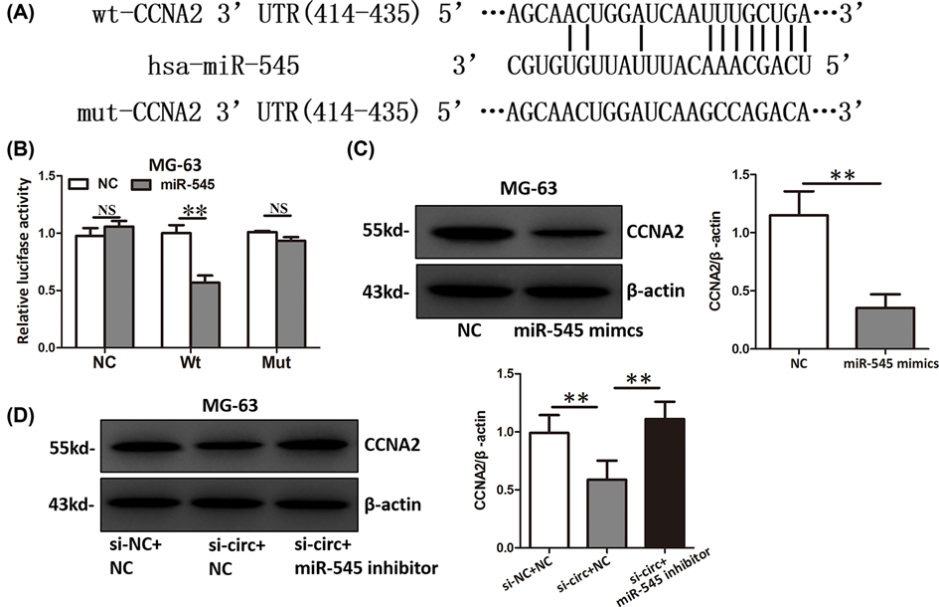
CCNA2 was a direct target of miR-545 in OS cells (**A**) Diagram showing the binding of miR-545 and CCNA2 3′UTR wild-type (Wt) or mutant (Mut). (**B**) Luciferase reporter assay showed miR-545 overexpression reduced the luciferase activity of the CCNA2 3′UTR Wt reporter but not the CCNA2 3′UTR Mut reporter. (**C**) CCNA2 protein level of MG-63 cells transfected with miR-545 mimics. (**D**) CCNA2 protein level of MG-63 cells co-transfected with hsa_circ_0003732 siRNA and miR-545 inhibitor. Data are presented as means ± S.D., NS, no significance, **P<0.01.

Through TargetScan and microRNA.org, we identified CCNA2 as a potential target of miR-545 (Figure 5A). To prove it, we performed luciferase reporter assay and found that miR-545 significantly inhibited the luciferase activity of MG-63 cells transfected with the reporter containing wild-type CCNA2 3′UTR but not the reporter vector containing the mutant binding sites of CCNA2 3′UTR (Figure 5B). Overexpression of miR-545 significantly downregulated the CCNA2 protein level (Figure 5C). Moreover, hsa_circ_0003732 knockdown up-regulated the CCNA2 protein level and this effect could be counteracted by inhibition of miR-545 (Figure 5D). Because CCNA2 is an oncogene in many cancers [14–16] and promote proliferation by regulating cell cycle, we think miR-545 might inhibit proliferation by suppressing CCNA2 expression in OS cells.

## Discussion

CircRNAs has been reported to be exert very important roles in human cancers, including OS [6–13]. Our study uncovered a novel aberrantly expressed circRNA, hsa_circ_0003732, in OS and this circRNA had never been studied either in OS or other cancers. So, we focused on hsa_circ_0003732 to study the clinical value, biological function and mechanism in OS.

In the present study, we first validated that hsa_circ_0003732 expression was significantly increased in OS tissues and cells. Further study showed that hsa_circ_0003732 expression was associated with tumor size and Enneking stage. High hsa_circ_0003732 expression was also correlated with low overall survival rates of OS patients. So, we assumed that hsa_circ_0003732 might function as an oncogene in OS and serve as a prognostic marker indicating poor outcome.

Then, we investigated the biological function of hsa_circ_0003732 in OS *in vitro*. We found that knockdown of hsa_circ_0003732 inhibited proliferation of OS cells. Mechanism research showed that hsa_circ_0003732 acted as a sponge for miR-545. MiR-545 was proved to be down-regulated and act as a tumor suppressor gene in many cancers, including gastric cancer [17], colorectal cancer [18], epithelial ovarian cancer [19], lung cancer [20] and oral squamous cell carcinoma [21]. We further demonstrated that miR-545 expression was also decreased in OS tissues and act as a tumor suppressor gene in OS. What’s more, we found a new target of miR-545, CCNA2, in OS. CCNA2 is an oncogene in cancers [14–16] and promote proliferation by regulating cell cycle and we showed that miR-545 might inhibit proliferation by suppressing CCNA2 expression in OS cells. Our results showed that hsa_circ_0003732 could regulate CCNA2 expression and then influence cell proliferation via miR-545. So, hsa_circ_0003732 could promote OS cells proliferation via miR-545/CCNA2 axis.

In conclusion, our study reveals that hsa_circ_0003732 is up-regulated and may serve as a potential prognostic marker in OS. Hsa_circ_0003732/miR-545/CCNA2 axis plays important roles in promoting OS cells proliferation and may be a potential therapeutic target for OS intervention.

## Abbreviations

ANT: adjacent noncancerous tissue
CCK-8: Cell Counting Kit-8
circRNA: circular RNA
EdU: 5-Ethynyl-2-deoxyuridine
OS: osteosarcoma
